# Diagnostic Potential of aMMP-8 PoCT in Oral Fluids: Insights from Periodontitis and Metabolic Syndrome Interplay

**DOI:** 10.1055/s-0045-1809977

**Published:** 2025-07-23

**Authors:** Julie Toby Thomas, Betsy Joseph, Timo Sorsa, Sukumaran Anil, Tuomas Waltimo

**Affiliations:** 1 Department of Oral and Maxillofacial Diseases, University of Helsinki and Helsinki University Hospital, Helsinki, Finland; 2 Department of Periodontics, Saveetha Dental College and Hospitals, Saveetha Institute of Medical and Technical Sciences, Chennai, Tamil Nadu, India; 3 Department of Dental Medicine, Karolinska Institutet, Stockholm, Sweden; 4 Faculty of Dentistry, Chulalongkorn University, Pathumwan, Bangkok, Thailand; 5 Global Research Cell, Dr. D. Y. Patil Dental College and Hospital, Dr. D. Y. Patil Vidyapeeth, Pimpri, Pune, India; 6 Faculty of Medicine, University of Basel, Basel, Switzerland

**Keywords:** metabolic syndrome, periodontal disease, active-matrix metalloproteinase 8, saliva, gingival crevicular fluid, oral rinse, ELISA

## Abstract

**Objective:**

This study evaluated the expression of active-matrix metalloproteinase-8 (aMMP-8) in saliva, oral rinse, and gingival crevicular fluid (GCF) of periodontitis patients with and without metabolic syndrome (MetS) and assessed correlations between aMMP-8 levels and both periodontal and metabolic parameters.

**Materials and Methods:**

One hundred and twenty participants aged 25 to 55 years were classified into three groups: MetS with periodontitis (MetS-PD, *n* = 40); systemically healthy with periodontitis (SH-PD, *n* = 40); and systemically and periodontally healthy controls (SH-PH, *n* = 40).

**Statistical Analysis:**

The median age of the participants was 49.5 ± 8.94 in MetS-PD, 47.5 ± 5.6 in SH-PD, and 42.5 ± 11.14 in SH-PH. Systemic parameters (waist circumference, blood pressure, lipid profile, and glycemic indices) and periodontal parameters (bleeding on probing, plaque score, probing depth, and clinical attachment loss) were assessed. aMMP-8 was evaluated qualitatively using point-of-care testing (PoCT) and quantitatively via ELISA in oral rinse, saliva, and GCF.

**Results:**

The PoCT was positive in 90% of MetS-PD and 85% of SH-PD participants, while all SH-PH participants tested negative ( *p* < 0.01). MetS-PD exhibited significantly higher aMMP-8 levels in oral rinse and saliva than SH-PD ( *p* = 0.001), whereas GCF aMMP-8 levels were higher in SH-PD but not substantially different from MetS-PD ( *p* = 0.086). Strong positive correlations were observed between aMMP-8 levels in all oral matrices and periodontal parameters ( *p* < 0.001). Among metabolic parameters, waist circumference, triglycerides, low-density lipoprotein, and fasting blood sugar showed stronger correlations with aMMP-8 levels than blood pressure measurements.

**Conclusion:**

The aMMP-8 PoCT effectively distinguished periodontally diseased individuals from healthy controls. Elevated aMMP-8 levels (>20 ng/mL) in oral fluids, especially saliva and oral rinse, were significantly associated with both periodontal and systemic risk factors, supporting the potential of aMMP-8 as a noninvasive biomarker for periodontitis and metabolic disorders.

## Clinical Relevance

*Rationale:* Active-matrix metalloproteinase-8 (aMMP-8) with a cut-off of 20 ng/mL is a promising biomarker for periodontal disease (PD) and systemic conditions such as metabolic syndrome (MetS). This study evaluated the point-of-care test (PoCT) qualitative and quantitative estimation of aMMP-8 in saliva, oral rinse, and gingival crevicular fluid (GCF) among different patient groups.

*Principal Findings:* aMMP-8 levels exceeding 20 ng/mL were found in oral rinse, saliva, and GCF of periodontitis groups compared to healthy controls ( *p* < 0.01). Oral rinse and saliva aMMP-8 levels were higher in MetS-PD than SH-PD, while GCF levels were higher in SH-PD. Strong correlations between aMMP-8 in oral fluids and periodontal parameters (bleeding on probing, probing pocket depth, clinical attachment loss) highlight its potential for noninvasive periodontal disease screening.

*Practical Implications:* The aMMP-8 biomarker and PoCT with a 20 ng/mL cut-off enable noninvasive early detection of periodontitis. The association between aMMP-8 and metabolic risk factors supports its utility in personalized treatment and preventive health care. Incorporating noninvasive sampling of saliva and oral rinse for aMMP-8 analysis into routine screenings can enhance early intervention and reduce disease burden.

## Introduction

Periodontal disease (PD), driven by dysbiotic biofilm, is a chronic inflammatory condition that causes irreversible destruction of alveolar bone and can lead to tooth loss. It is often linked to systemic diseases like diabetes and obesity, which are components of metabolic syndrome (MetS)—a cluster of conditions that increases the risk of cardiovascular disease, diabetes, and stroke. 1 The progression of periodontitis is influenced by systemic diseases and, when unmonitored, can potentially worsen ongoing periodontal destruction, leading to increased cardiovascular risk and other systemic complications. Early detection and intervention are crucial to address the global burden of periodontitis and its association with systemic diseases.

Matrix metalloproteinase-8 (MMP-8), a major collagenase-2 or neutrophil collagenase primarily degranulated by neutrophils, accounts for 80% of collagenase activity in gingival crevicular fluid (GCF). Proinflammatory cytokines such as tumor necrosis factor-α (TNF-α), interleukin-1β, reactive oxygen species, and proteases originating from the host in response to virulence factors produced by subgingival biofilm like lipopolysaccharides can release and/or activate MMP-8 during periodontal inflammation, further causing irreversible periodontal destruction. 2 It has been reported that *Treponema denticola* (Td)-dentilisin in chronic periodontitis gingivae is linked to elevated aMMP-8 (active MMP-8) levels, increasing the risk of periodontal tissue collagenolysis and periodontitis progression. 3 Increased levels of MMP-8 in its catalytically active collagenolytic form in GCF tend to rise in clinically progressive active sites of severe periodontitis patients, whereas the total or latent non-collagenolytic pro-form of MMP-8 (total MMP-8) declines, making the active form a robust significant biomarker for diagnosing and monitoring periodontitis. It is important to distinguish between aMMP-8 and tMMP-8 as periodontitis biomarkers.

Periodontics focuses on using advanced science for diagnosing and treating PDs, which are costly and time-consuming to manage. Early detection improves treatment planning and disease monitoring during maintenance phases. Oral fluids including GCF, saliva, and oral rinse are gaining attention for diagnosis and monitoring of PD. Interestingly, elevated aMMP-8 levels—but not tMMP-8—in GCF, saliva, and oral rinse have been found to distinguish periodontitis from gingivitis and predict attachment loss. 4 5 6 Recent studies investigating the expression and levels of MMP-8 in different biofluids including serum, saliva, GCF, and oral rinse have reported that biomarker levels are influenced by age, gender, risk factors like tobacco use, type 2 diabetes mellitus, and MetS, suggesting the need for further considerations in future epidemiological studies. 7 8 9 10 11

Researchers are focused on identifying suitable oral fluid sampling methods that offer fast, actionable results at the point of care (PoC), reducing time, resources, and costs compared to traditional methods for early disease detection. Despite GCF being a significant biological fluid for oral health research, various challenges are encountered with respect to its limited volume, contamination risk, and site-specific nature, making it difficult to generalize results across many sites or individuals. 12 Standardized protocols for collection, processing, and analysis make saliva samples more cost-effective, especially in resource-limited settings or large-scale epidemiological studies. Saliva also reflects both local oral conditions and systemic health, making it valuable for detecting biomarkers of systemic diseases.

Noninvasive aMMP-8 PoC diagnostic tests using oral rinse have consistently identified active periodontal tissue destruction and have been effective in predicting disease progression and monitoring treatment outcomes. However, the varied composition and properties of different oral fluid matrices may affect biomarker profiles and test results when applied in diagnostics. Since most existing approaches are observational and little is known about how various sampling techniques affect accuracy, a proven and efficient process must be developed. In line with previous findings, we hypothesize that the expression of aMMP-8 in oral rinse is comparable with that found in saliva and GCF and could be utilized as a diagnostic fluid to discriminate periodontitis among MetS and systemically healthy populations.

This study aims to: (1) compare and investigate the expression and levels of aMMP-8 in different oral fluids (oral rinse, saliva, and GCF) based on their unique abilities to identify site-specific versus systemic inflammatory responses; (2) assess its diagnostic efficiency to discriminate among healthy individuals and periodontitis patients with and without MetS; and (3) identify correlations between aMMP-8 levels in oral fluids with clinical periodontal parameters (bleeding on probing [BoP], periodontal pocket depth, and clinical attachment loss [CAL]) and metabolic parameters.

## Materials and Methods

### Study Design and Study Population

This cross-sectional study was conducted among Indian adults aged 25 to 55 years from June 15, 2023 to December 15, 2023 at the Pushpagiri Institute of Medical Sciences and Research Centre in Kerala, India. The research was approved by the Institutional Review Board of the Pushpagiri Institute of Medical Sciences and Research Center on May 5, 2023 (IRB Study Reference No: 20/0112023). The study was conducted in accordance with the revised Declaration of Helsinki and reported following the STROBE (Strengthening the Reporting of Observational Studies in Epidemiology) guidelines.

The following formula was used to determine the sample size ( *n* ):



With *Z _α_*_/2_ = 1.96 and *Z*_ 1 − *β*_ = 1.28 representing the 95% confidence values from a standard normal distribution, a minimum of 40 subjects was needed to detect a moderate difference in salivary biomarkers. This calculation was based on an effect size of 300 and a standard deviation of 414.17 from a pilot survey. Consequently, the final required sample size was 40 participants per group.

One hundred and twenty participants from the Periodontology and Dental Outpatient Department, Pushpagiri Institute of Medical Sciences and Research Center, Kerala were selected by systematic random sampling. The primary investigator (J.T.T.) conducted basic periodontal screening using a WHO (World Health Organization) probe. 13 Participants with a score of 4 (probing depth >5.5 mm in at least two sextants) underwent detailed periodontal examinations by two investigators (J.T.T. and B.J.). The participants with severe periodontitis (stage III/IV) with grade B/C were diagnosed based on the following criteria: ≥5 mm CAL, ≥6 mm probing pocket depth (PPD) at ≥2 nonadjacent teeth with radiographic bone loss extending ≥ middle third of the teeth affected. Indirect assessment of the grading was done by dividing the % of bone loss of the most affected site by age of the participant. 14 Participants with BoP <10%, PPD ≤3 mm, and absence of CAL were considered periodontally healthy. 15

Participants were classified based on the presence or absence of MetS, with 40 individuals recruited per group. Diagnosis followed the 2006 International Diabetes Federation criteria 16 requiring increased waist circumference (WC) (≥90 cm for men, ≥80 cm for women) along with at least two of the following: elevated triglycerides (TGs; ≥150 mg/dL), reduced high-density lipoproteins (HDL; <40 mg/dL in men, <50 mg/dL in women), high blood pressure (≥130/85 mmHg), or fasting blood sugar (FBS) ≥100 mg/dL.

**Fig. 1:**
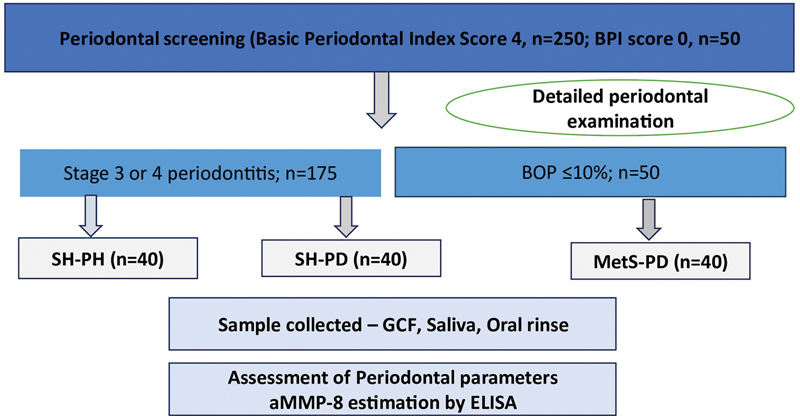
Flowchart on patient allocation.

A control group ( *n* = 40) included systemically and periodontally healthy individuals attending for routine dental care. Exclusion criteria encompassed systemic diseases (other than MetS), extension of CAL less than 30% of the teeth, recent periodontal treatment or antibiotics (past 6 months), <20 teeth, bisphosphonate use, nonsteroidal anti-inflammatory drug/corticosteroid therapy, smoking, pregnancy/lactation, or lack of informed consent. Fig. 1 illustrates a flowchart on patient allocation.

The study groups recruited included:

MetS-PD: 40 participants with MetS and severe periodontitis.
SH-PD: 40 systemically healthy participants with severe periodontitis.
SH-PH: 40 systemically healthy participants with a healthy periodontium.

### Assessment of Variables

#### Sociodemographic, Oral Hygiene, and Systemic Parameters

A validated, closed-ended questionnaire was used to gather sociodemographic information, including place of residence, age, gender, education level, medical history, smoking habits, past dental history, and routine oral hygiene practices ( **Appendix 1** : Sociodemographic and Oral Hygiene Questionnaire). Following this, a physical examination of all consenting participants was conducted, along with recording of systemic parameters including systolic and diastolic blood pressure (SBP and DBP) and WC. Blood pressure was measured after 5 to 10 minutes of rest from the right arm using a digital sphygmomanometer (Omron HEM 7120, Vietnam), with subjects seated and backs supported. The average of two readings was recorded. Additionally, after 12 to 14 hours of fasting, blood samples were collected by the third investigator (B.J.) to estimate serum biochemical parameters, including TG, HDL, low-density lipoprotein (LDL), and fasting blood glucose.

#### Clinical Periodontal Parameters

Periodontal assessments were conducted by two calibrated investigators (J.T.T. and B.J.), who received training from a specialist in periodontology (S.A.) before undergoing inter-examiner calibration. Calibration was performed on 10 participants at different time intervals for all periodontal indices. Inter-examiner reliability was assessed using Cohen's kappa, with scores ≥0.85 achieved for all parameters, indicating excellent agreement.

The periodontal parameters assessed for each participant included the percentage of sites with BoP, 17 PPD, CAL, and the full-mouth plaque score (FMPS), determined by the percentage of plaque-positive sites. 18

The periodontal parameters assessed for each participant included the percentage of sites with BoP, 17 PPD, CAL, and the FMPS, which was determined by the percentage of plaque-positive sites. Measurements were obtained at six specific sites per tooth (mesio-buccal, mesio-lingual, mid-buccal, disto-buccal, disto-lingual, and mid-lingual) using a UNC-15 probe (Hu-Friedy, United States). BoP was recorded as either present or absent within 10 seconds of probing. PPD was measured from the free gingival margin to the base of the pocket, while CAL was determined from the cementoenamel junction to the bottom of the pocket or sulcus. 19

#### Biochemical Analysis of Human Active MMP-8 Using ELISA Kit

Study participants were instructed to abstain from food, fluids, and chewing gum for 1 hour before saliva collection and to report between 10:00 a.m. and 12:00 p.m. to minimize diurnal variation. Sample collection was performed by investigators blinded to patient group allocation. Unstimulated saliva was collected from each participant into 5 mL sterile Falcon tubes (Maxome Labsciences, Bengaluru, India) using the spitting method, following published guidelines. 20 Participants sat upright for 5 minutes, expectorating into sterile tubes every 60 seconds. The oral rinse sampling was conducted by the same examiner (J.T.T.) using the following protocol: (1) prerinsing the oral cavity with tap water, followed by a 30-second rinse with 5 mL purified water provided in the test kit; and (2) pouring the oral rinse into a collection cup. A commercial lateral flow mouth rinse immunoassay test (PerioSafe, Dentognostics GmbH) was used for qualitative analysis of aMMP-8 with a cut-off of 20 ng/mL in oral rinse samples. 21

GCF samples were collected by inserting periostrips (Perio-paper, IDE Interstate, Amityville, New York, United States) into the two deepest periodontal pocket sites of nonadjacent teeth per patient. Prior to sampling, the crown of the selected tooth was gently cleaned, dried, and isolated with cotton rolls to prevent saliva contamination. A standard paper strip was inserted into the sulcus to a depth of 1 to 2 mm for 30 seconds. Strips contaminated by blood were discarded, and GCF was collected from different sites of nonadjacent teeth. 22 To prevent interference with GCF quality and quantity, clinical measurements were recorded after GCF sampling. The sampling strips were placed into test tubes containing 0.5 mL of HEPES-buffer (pH 7.4), and phosphate-buffered saline was used to elute GCF from the periostrips. All oral samples were stored at −20°C until analysis.

##### Quantitative Analysis of Active MMP-8

The stored samples at −20°C were subjected to quantitative sandwich ELISA (enzyme-linked immunosorbent assay) analysis for aMMP-8 23 using commercially available ELISA kits which were procured for Human active MMP-8 (Catalog No: EK0464, Boster Bio).

In addition, 100 μL of oral samples and standards were added to wells precoated with MMP-8 antibody and incubated at 37°C for 60 minutes. The sample was aspirated, and 100 μL of biotinylated antibody was added, followed by incubation at 37°C for 60 minutes. Wells were aspirated and washed twice with wash buffer, and then 100 μL of HRP-conjugated streptavidin was added and incubated for 30 minutes at 37°C. After aspiration and repeat washing, 100 μL of TMB substrate was added and incubated for 10 minutes at 37°C. The reaction was stopped by adding 100 μL of stop solution, changing the color from blue to yellow. Optical density was read at 450 nm within 10 to 15 minutes.

### Statistical Analysis

Statistical analyses were performed using IBM SPSS version 26.0 (IBM, Chicago, Illinois, United States). Descriptive and categorical variables were summarized as frequencies and percentages. Continuous variables were summarized as means and standard deviations. Based on the statistical central limit theorem, parametric tests were conducted for each group since the sample size exceeded 30 per group. One-way ANOVA (analysis of variance) was used to analyze group differences, with Scheffe's post-hoc tests for inter-group comparisons. Pearson correlation analysis was performed to evaluate the relationship between aMMP-8 levels in oral rinse, saliva, and GCF with clinical periodontal parameters (BoP, PPD, CAL) and metabolic parameters. A *p* -value <0.05 was considered statistically significant.

## Results

### Demographic Distribution and Oral Hygiene Practices of the Participants

One hundred and twenty participants were categorized into three groups: MetS-PD ( *n* = 40), SH-PD ( *n* = 40), and SH-PH ( *n* = 40). The median age was highest in the MetS-PD group (49.5 ± 8.94 years), followed by the SH-PD group (47.5 ± 5.6 years), and lowest in the SH-PH group (32.5 ± 11.14 years). Females constituted the majority in all three groups, with the highest percentage in the SH-PD group (82.5%). Males were more evenly distributed among the MetS-PD and SH-PH groups (32.5% each). Participants were approximately equally distributed between rural and urban areas. The highest percentage of graduate/postgraduate participants was found in the MetS-PD group (37.5%), followed by the SH-PH (30%) and SH-PD (22.5%) groups, though no significant differences were found among the groups regarding educational qualification.

The frequency of tooth brushing did not differ significantly across groups ( *p* = 0.52), with most participants brushing once daily. The majority used a toothbrush and toothpaste, with minimal use of charcoal powder or toothbrush alone ( *p* = 0.67). Self-reported halitosis was significantly more frequent in the MetS-PD group compared to the SH-PD and SH-PH groups ( *p* < 0.01). Similarly, bleeding on brushing was more common in MetS-PD than in the other groups ( *p* < 0.01). Dental visits were infrequent across all groups, with most participants visiting only occasionally ( *p* = 0.549; Table 1 ).

**Table 1:** Demographic and oral hygiene practices among the participants of different groups

Variables		MetS-PD ( *N =* 40)	SH-PD ( *N =* 40)	SH-PH ( *N =* 40)	*p-* Value
Age (y)	Median ± SD	49.5 ± 8.94	47.5 ± 5.6	32.5 ± 11.14	0.116
Gender	Male, *n* (%)	13 (32.5)	7 (17.5)	13 (32.5)	0.222
	Female, *n* (%)	27 (67.5)	33 (82.5)	27 (67.5)	
Place of residence	Rural, *n* (%)	20 (50.0)	19 (47.5)	21 (52.5)	0.905
	Urban, *n* (%)	20 (50)	21 (52.5)	19 (47.5)	
Educational qualification	Graduate/postgraduate, *n* (%)	15 (37.5)	9 (22.5)	12 (30)	0.604
	Completed high school, *n* (%)	19 (47.5)	23 (57.5)	23 (57.5)	
	Completed primary school/illiterate, *n* (%)	6 (15)	8 (20)	5 (12.5)	
Frequency of brushing	Once a day	26 (65)	28 (70)	31 (77.5)	0.52
	Twice a day	14 (35)	12 (30)	9 (22.5)	
Method of cleaning teeth	Use only toothbrush	1(2.5)	0 (0)	0 (0)	0.67
	Toothbrush and paste	36 (90)	38 (95)	38 (95)	
	Use of charcoal power	3 (7.5)	2 (5)	2 (5)	
Complaint of halitosis	Often	6 (15)	2 (5)	0 (0)	<0.01 a
	Sometimes	21 (52.5)	4 (10)	8 (20)	
	Occasional/never	12 (32.5)	34 (85)	32 (80)	
Bleeding on brushing	Often	3 (7.5)	1(2.5)	0 (0)	<0.01 a
	Sometimes	23 (57.5)	6 (15)	14 (35)	
	Occasional/never	14 (35)	33 (82.5)	26 (65)	
Frequency of dental visit	Once in a year	2 (5)	4 (10)	2 (5)	0.549
	Occasional	37 (92.5)	36 (90)	38 (95)	
	Never	1 (2.5)	0 (0)	0 (0)	

Abbreviation: SD, standard deviation.

Note: Values are expressed as median ± standard deviation and number (percentage). One-way analysis of variance (ANOVA) followed by post-hoc Tukey test comparisons was used for continuous variables.

a Statistically significant at 1% level ( *p* < 0.01).

MetS-PD participants had significantly higher WC, blood pressure, TG, LDL, and FBS compared to SH-PD and SH-PH groups ( *p* < 0.01), confirming their MetS status. HDL levels showed no significant differences between groups ( *p* = 0.551). Regarding periodontal parameters, BoP was highest in the MetS-PD group (56.68 ± 25.41%) and lowest in the SH-PH group (7.99 ± 1.72%) ( *p* < 0.01). FMPS was similar between MetS-PD and SH-PD groups but significantly lower in the SH-PH group ( *p* < 0.01). PPD and CAL were most severe in the MetS-PD group (6.70 ± 1.42 mm and 8.60 ± 2.04 mm, respectively) and least severe in the SH-PH group (3.03 ± 0.53 mm and 0.75 ± 1.32 mm, respectively) ( *p* < 0.01). The number of missing teeth was highest in the MetS-PD group (4.05 ± 2.60) and lowest in the SH-PH group (0.15 ± 0.66) ( *p* < 0.01; Table 2 ).

**Table 2:** Systemic and periodontal parameters among the participants of different groups

Variables	MetS-PD ( *N =* 40)		SH-PD ( *N =* 40)		SH-PH ( *N =* 40)		*p* -Value
	Mean	SD	Mean	SD	Mean	SD	
WC (cm)	107.10 ^a^	11.13	97.14 ^b^	9.44	91.88 ^c^	13.67	<0.01**
SBP (mm of Hg)	130.00 ^a^	15.94	114.55 ^b^	10.50	116.75 ^b^	11.18	<0.01**
DBP (mm of Hg)	85.98 ^a^	10.35	76.90 ^b^	7.45	78.15 ^b^	5.60	<0.01**
TG (mg/dL)	133.70 ^a^	30.47	102.60 ^b^	30.92	97.18 ^b^	34.43	<0.01**
LDL (mg/dL)	140.73 ^a^	52.32	120.95 ^b^	30.79	89.85 ^c^	18.05	<0.01**
HDL (mg/dL)	57.86 ^a^	10.66	56.75 ^a^	8.60	54.93 ^a^	15.90	0.551
FBS (mg/dL)	148.38 ^a^	37.29	102.10 ^b^	12.12	97.05 ^b^	28.05	<0.01**
BoP (%)	56.68 ^a^	25.41	45.45 ^b^	27.53	7.99 ^c^	1.72	<0.01**
FMPS (%)	39.18 ^a^	16.75	38.40 ^a^	21.14	23.05 ^b^	12.78	<0.01**
PPD (mm)	6.70 ^a^	1.42	5.80 ^b^	1.22	3.03 ^c^	0.53	<0.01**
CAL (mm)	8.60 ^a^	2.04	7.63 ^b^	1.86	0.75 ^c^	1.32	<0.01**

Abbreviations: BoP, bleeding on probing; CAL, clinical attachment loss; DBP, diastolic blood pressure; FBS, fasting blood sugar; FMPS, full mouth plaque score; HDL, high-density lipoprotein; LDL, low-density lipoprotein; PPD, probing pocket depth; SBP, systolic blood pressure; SD, standard deviation; TG, triglycerides; WC, waist circumference.

Note: One-way ANOVA test was used for continuous variables to analyze the differences across the three groups, and Scheffe's post-hoc test was used for pairwise difference between the groups. Values with different superscripted letters indicate a statistically significant pairwise difference ( *p* < 0.05) by Scheffe's post-hoc test. Values with the same superscripted letters indicate a statistically significant pairwise difference ( *p* > 0.05).

** Statistically significant at 1% level ( *p* < 0.01).

### Comparison of aMMP-8 in Oral Matrices

**Fig. 2:**
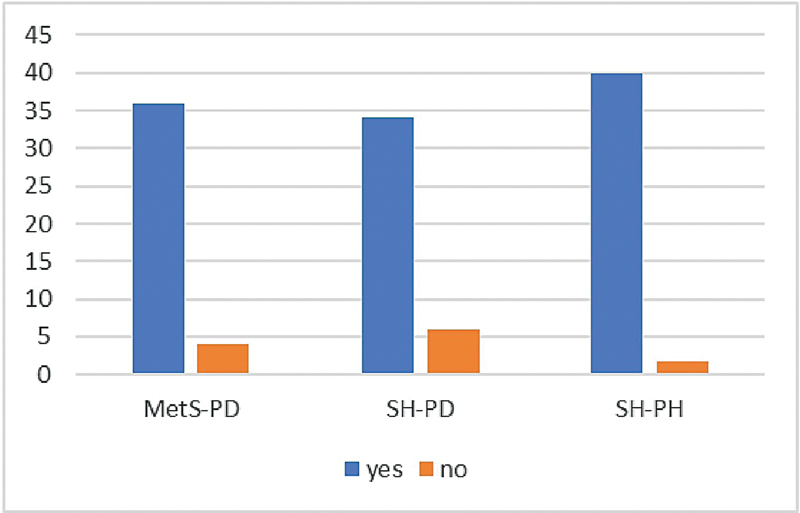
Comparison of the results of aMMP-8 PoC test.

Fig. 2 depicts the qualitative estimation of aMMP-8 using PoC test (PoCT) with cut-off of 20 ng/mL using mouthrinse among the three groups. The aMMP-8 PoCT was positive in 90% >20 ng/mL of MetS-PD and 85% of SH-PD participants, while all SH-PH participants tested negative (<20 ng/mL; *p* < 0.01).

**Fig. 3:**
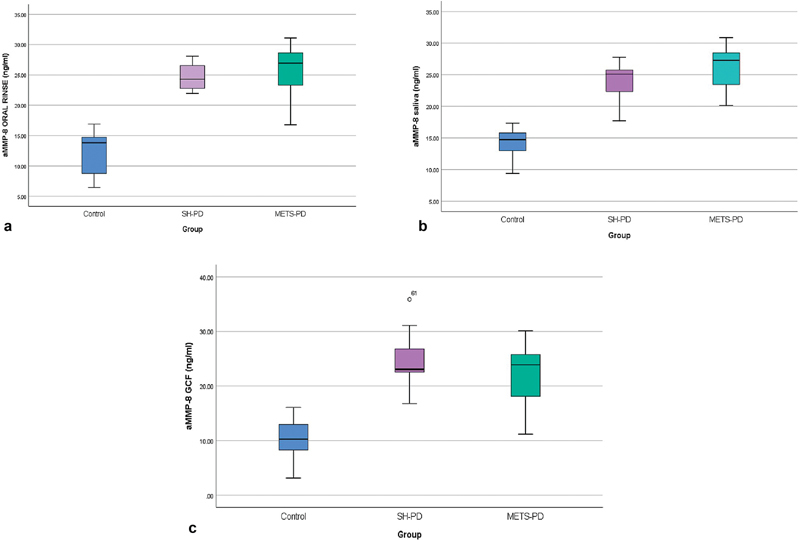
Comparison of aMMP-8 levels in different oral fluids in each group: **(a)** oral rinses; **(b)** saliva; **(c)** GCF. GCF, gingival crevicular fluid.

The mean aMMP-8 levels in oral rinse, saliva, and GCF varied significantly across the control, SH-PD, and MetS-PD groups ( *p* < 0.01, one-way ANOVA; Table 3 and Fig. 3 ). The control group consistently had the lowest levels (less than 20 ng per mL) across all sample types (oral rinse, saliva, and GCF). For all sample types (oral rinse, saliva, and GCF), statistically significant differences in aMMP-8 levels were observed between the control and both the diseased groups, SH-PD and METS-PD groups ( *p* < 0.01). Though not significant in oral rinse ( *p* = 0.433), the METS-PD group showed consistently higher mean aMMP-8 levels in oral rinse and saliva compared to SH-PD (26.25 vs. 24.10 ng/mL, *p* = 0.001).

**Table 3:** Comparison of aMMP-8 levels in different oral fluids across different groups

Variables	MetS-PD ( *N =* 40)		SH-PD ( *N =* 40)		SH-PH ( *N =* 40)		*p* -Value
	Mean	SD	Mean	SD	Mean	SD	
aMMP-8 Oral rinse (ng/mL)	25.66 ^a^	4.29	24.68 ^a^	2.00	12.17 ^b^	3.40	<0.01**
aMMP-8 saliva (ng/mL)	26.25 ^a^	3.00	24.10 ^b^	2.56	14.36 ^c^	1.89	<0.01**
aMMP-8 GCF (ng/mL)	22.07 ^a^	4.82	24.13 ^a^	4.17	10.31 ^b^	3.19	<0.01**

Abbreviations: aMMP-8, active-matrix metalloproteinase-8; GCF, gingival crevicular fluid; SD, standard deviation.

Note: One way ANOVA test was used for continuous variables to analyze the differences across the three groups, and Scheffe's post-hoc test was used for pairwise difference between the groups. Values with different superscripted letters indicate a statistically significant pairwise difference ( *p* <0.05) by Scheffe's post-hoc test. Values with the same superscripted letters indicate a statistically significant pairwise difference ( *p* >0.05).

**Statistically significant at 1% level ( *p* < 0.01).

Alternatively, aMMP-8 levels in GCF were higher in SH-PD compared to the METS-PD group, but the findings were not statistically significant (24.13 vs. 22.07 ng/mL, *p* = 0.086). Recall that SH-PH aMMP-8 levels in all fluid matrices (oral rinse, saliva, and GCF) were without exception <20 ng/mL.

### Correlation between Periodontal Parameters and aMMP-8 Levels in Oral Matrices

Results summarized in Table 4 demonstrate significant positive correlations between aMMP-8 levels in all three oral matrices (oral rinse, saliva, and GCF) and periodontal parameters, including BoP, PPD, and CAL ( *p* < 0.001 for all correlations).

**Table 4:** Correlation between periodontal parameters and aMMP-8 levels in oral matrices

aMMP-8 in oral matrices ( *N =* 120)	BOP (%)	PPD (mm)	CAL (mm)
aMMP-8 Oral rinse (ng/mL)	0.559**	0.684**	0.770**
aMMP-8 Saliva (ng/mL)	0.601**	0.705**	0.776**
aMMP-8 GCF (ng/mL)	0.546**	0.642**	0.743**

Abbreviations: aMMP-8, active-matrix metalloproteinase-8; BOP, bleeding on probing; CAL, clinical attachment loss; GCF, gingival crevicular fluid; PPD, periodontal pocket depth.

**Statistically significant at 1% level ( *p* < 0.01).

aMMP-8 in oral rinse showed a moderate correlation with BoP ( *r* = 0.559) and stronger correlations with PPD ( *r* = 0.684) and CAL ( *r* = 0.770). aMMP-8 in saliva exhibited slightly stronger correlations with BoP ( *r* = 0.601), PPD ( *r* = 0.705), and CAL ( *r* = 0.776) compared to oral rinse. In contrast, aMMP-8 in GCF demonstrated relatively weaker correlations among the three matrices, although still statistically significant, with BoP ( *r* = 0.546), PPD ( *r* = 0.642), and CAL ( *r* = 0.743).

### Correlation between Systemic Parameters and aMMP-8 Levels in Oral Matrices

Analysis of the relationships between systemic parameters and aMMP-8 levels revealed several significant correlations ( Table 5 ). A significant positive correlation was observed between WC and aMMP-8 levels in all matrices (oral rinse: *r* = 0.34, *p* < 0.01; saliva: *r* = 0.33, *p* < 0.01; GCF: *r* = 0.34, *p* < 0.01), indicating an association between central adiposity and elevated aMMP-8 expression.

**Table 5:** Correlation between metabolic parameters and aMMP-8 levels in oral matrices

Parameters ( *N =* 120)	aMMP-8 Oral rinse (ng/mL)	aMMP-8 Saliva (ng/mL)	aMMP-8 GCF (ng/mL)
WC (cm)	0.34**	0.33**	0.34**
SBP (mm of Hg)	0.16NS	0.19*	0.07NS
DBP (mm of Hg)	0.18*	0.19*	0.01NS
TG (mg/dL)	0.27**	0.28**	0.13NS
LDL (mg/dL)	0.44**	0.43**	0.27**
HDL (mg/dL)	0.04NS	0.135NS	0.07NS
FBS (mg/dL)	0.34**	0.371**	0.27**

Abbreviations: aMMP-8, active-matrix metalloproteinase; DBP, diastolic blood pressure; FBS, fasting blood sugar; HDL, high-density lipoprotein; LDL, low-density lipoprotein; SBP, systolic blood pressure; TG, triglycerides; WC, waist circumference.

*Significant at 5% level ( *p* < 0.05); NS: nonsignificant.

**Statistically significant at 1% level ( *p* < 0.01).

Regarding blood pressure parameters, SBP correlated significantly with aMMP-8 levels in saliva ( *r* = 0.19, *p* < 0.05), but the correlation was nonsignificant in oral rinse ( *r* = 0.16, NS) and GCF ( *r* = 0.07, NS). DBP was significantly associated with aMMP-8 in both oral rinse ( *r* = 0.18, *p* < 0.05) and saliva ( *r* = 0.19, *p* < 0.05), but not in GCF ( *r* = 0.01, NS).

Among lipid parameters, HDL showed significant positive correlations with aMMP-8 in oral rinse ( *r* = 0.27, *p* < 0.01) and saliva ( *r* = 0.28, *p* < 0.01), but not in GCF ( *r* = 0.13, NS). LDL levels were strongly correlated with aMMP-8 across all matrices, with the highest correlation observed in oral rinse ( *r* = 0.44, *p* < 0.01), followed by saliva ( *r* = 0.43, *p* < 0.01) and GCF ( *r* = 0.27, *p* < 0.01). In contrast, HDL did not show significant correlations with aMMP-8 in any matrix.

FBS demonstrated significant positive correlations with aMMP-8 across all matrices, with the strongest association in saliva ( *r* = 0.371, *p* < 0.01), followed by oral rinse ( *r* = 0.34, *p* < 0.01) and GCF ( *r* = 0.27, *p* < 0.01).

## Discussion

Catalytically competent collagenolytic aMMP-8 (neutrophil collagenase or collagenase-2) has emerged as a valuable biomarker for predicting, diagnosing, and monitoring periodontitis and peri-implantitis. 21 24 25 Its ability to reflect real-time tissue destruction makes it an essential tool for early intervention and personalized periodontal care. 25 26 27 28 Despite its potential, challenges related to sample collection, assay repeatability, and standardization remain in MMP-8 detection. PoC MMP-8 diagnostics have the potential to revolutionize PD management through enabling early detection, real-time monitoring, and targeted treatment strategies. 29 The present study evaluated aMMP-8 levels in three different oral fluids—oral rinse, saliva, and GCF—and assessed their diagnostic efficacy in distinguishing between healthy individuals, systemically healthy periodontitis patients, and those with MetS and periodontitis. Additionally, we examined correlations between aMMP-8 levels and both periodontal and metabolic parameters to better understand the interplay between local and systemic factors in periodontitis.

### aMMP-8 Levels in Different Oral Matrices

Our results demonstrate that aMMP-8 levels were significantly elevated in all three oral fluids in periodontitis patients (both with and without MetS) compared to healthy individuals. This confirms the diagnostic utility of aMMP-8 as a biomarker for PD. Interestingly, the highest expression in the periodontitis groups was observed in saliva, followed by oral rinse and GCF. These findings align with Katsiki et al, 30 who reported significantly higher biomarker concentrations in oral rinse samples of periodontitis patients compared to controls.

Our study confirms and further extends the well-established link between aMMP-8 and PD, demonstrating its positive correlation with clinical markers like BoP, PPD, and CAL. However, a novel finding is that MetS appears to amplify aMMP-8 expression in saliva and oral rinse, but not in GCF. 11 31 This elevation in MetS-PD patients may result from systemic inflammation characterized by increased pro-inflammatory cytokines like interleukin-6 and TNF-α, as well as insulin resistance, which collectively contribute to enhanced MMP activity, oxidative stress, and periodontal tissue breakdown. 2 This observation aligns with previous reports describing the bidirectional relationship between PD and MetS 32 and highlights aMMP-8 as a potential biomarker linking metabolic dysregulation with periodontitis. This underscores the multifactorial nature of periodontitis as influenced by systemic health, particularly in conditions like diabetes. 7 8 9 10 11

### Cut-off Values and Diagnostic Utility

Previous research has established an optimal cut-off level of 20 ng/mL in mouth rinse for the aMMP-8 PoCT as the most effective threshold for distinguishing between periodontal health and disease across different ethnic cohorts globally. 3 10 11 29 This has been successfully implemented as a well-functioning biomarker test in Tonetti et al's new staging and grading classifications of periodontitis and peri-implantitis. 11 25 33 34 In our study, aMMP-8 levels in the control group (SH-PH) were consistently below 20 ng/mL in all three oral fluids, which was further substantiated by the visual outcomes of the PoCT using oral rinse. The comparable levels of aMMP-8 in oral rinse relative to saliva and GCF from the same patients—reflecting disease severity—support its potential utilization as a noninvasive and easily obtainable diagnostic sample for periodontitis diagnosis and monitoring. Furthermore, our findings confirm that aMMP-8 PoCT with a cut-off of 20 ng/mL in oral fluid matrices can effectively serve as a biomarker to assess periodontitis and peri-implant health. 3 10 11 24 27 29

### Comparative Analysis of Oral Matrices

The analysis of aMMP-8 levels across different oral fluids revealed important distinctions. While all three matrices showed elevated aMMP-8 in periodontitis patients compared to healthy controls, there were notable differences between the MetS-PD and SH-PD groups. In oral rinse and saliva, the MetS-PD group demonstrated higher aMMP-8 levels, whereas in GCF, the SH-PD group showed higher levels, although the difference was not statistically significant. This pattern reinforces findings from our previous study 11 and highlights the diagnostic potential of aMMP-8 in oral fluids for identifying not only PD but also for screening patients with early metabolic disturbances. 10 35

Oral rinse aMMP-8 testing is optimized to detect enzymes originating from GCF, as vigorous rinsing facilitates the release of GCF into the oral cavity. Advanced oral fluid-based methods such as IFMA and dentoELISA, which employ antibodies specific to the active forms of MMP-8 derived from neutrophils and fibroblasts, have shown superior diagnostic performance compared to conventional ELISA. These methods selectively identify the biologically active MMP-8 isotypes that are abundant in GCF during active periodontitis. 23 25 While these active forms are localized to GCF, oral rinse samples effectively capture them due to mechanical mobilization during swishing. Notably, our study found that oral rinse and saliva samples could distinguish individuals with systemic disease coexisting with periodontitis from those with periodontitis alone, demonstrating their ability to reflect both local periodontal inflammation and broader systemic inflammatory conditions. In contrast, GCF, due to its site-specific nature, lacked this discriminatory power. These observations are consistent with prior research supporting oral rinse aMMP-8 testing as a noninvasive, whole-mouth screening tool that encompasses both periodontal and systemic health indicators. 36

The observed higher aMMP-8 levels in GCF of SH-PD compared to MetS-PD (though not statistically significant) might be attributed to medication effects in the MetS group. Many participants with MetS were taking medications such as statins, which could attenuate periodontal inflammation and enzyme expression in site-specific fluid parameters. 37 38 Additionally, the standardized GCF sampling protocol with limited time confinement might have resulted in decreased aMMP-8 levels compared to the increased expression observed in oral rinse and saliva. The broader standard deviations in aMMP-8 levels observed in the MetS-PD group across all sample types indicate greater heterogeneity, possibly due to factors such as glycemic control and medication use influencing MMP-8 concentrations in patients with metabolic disturbances. 39

### Correlations with Clinical Parameters

Our observation of strong positive correlations between aMMP-8 levels in all oral matrices and key periodontal parameters (BoP, PPD, and CAL) is consistent with previous literature indicating that elevated aMMP-8 levels correlate with active periodontal destruction. 4 11 Previous studies have identified higher MMP-8 expression in GCF due to its site-specific nature, whereas biomarker levels in other biofluids like oral rinse and saliva may be diluted. 40 Interestingly, in our study, GCF showed a relatively weaker correlation with periodontal parameters compared to saliva and oral rinse. This unexpected finding could be attributed to the limited sample volume procured and standardization challenges. Nevertheless, GCF still holds value as a complementary marker in more targeted clinical settings where deeper periodontal pockets need to be monitored.

The consistency of aMMP-8 levels across various sampling methods—particularly oral rinse and saliva—indicates the potential of these less invasive options to substitute for GCF in certain diagnostic contexts. The decision regarding patient-specific versus site-specific aMMP-8 testing should be based on individual clinical circumstances and diagnostic objectives. Saliva, with its strong correlations with periodontal metrics (especially CAL), proves effective for monitoring inflammation and early disease detection. Its ease of collection enhances its suitability for PoC diagnostics, aligning with trends in personalized health care. While oral rinse also effectively measures aMMP-8, its diagnostic sensitivity is slightly lower than that of saliva, potentially making it more suitable for general screening of conditions like MetS and diabetes rather than detailed monitoring in severe periodontal cases. 11 41 42 43 44

### Relationship with Metabolic Parameters

Our correlation analysis revealed significant associations between aMMP-8 levels and several metabolic parameters. WC, TG, LDL cholesterol, and FBS showed particularly strong correlations with aMMP-8 levels in oral rinse and saliva, highlighting the potential systemic influence on this biomarker. These findings are consistent with our previous results in saliva 11 and suggest that aMMP-8 may serve as a link between periodontal inflammation and metabolic dysregulation. Interestingly, aMMP-8 levels in GCF were less responsive to TG and SBP/DBP, suggesting that this site-specific fluid may be more reflective of local inflammatory processes than systemic metabolic disturbances. This differential pattern across oral matrices underscores the importance of selecting appropriate sampling methods based on the specific diagnostic objectives.

### Strengths and Limitations

A major strength of this study is the comparative analysis of multiple oral matrices (saliva, oral rinse, GCF) for aMMP-8 assessment, enhancing the robustness of our findings. The noninvasive nature of aMMP-8 testing in oral rinse makes it a practical tool for large-scale screening and early detection of periodontitis, particularly among high-risk individuals with MetS.

The inclusion of both qualitative PoCT and quantitative ELISA measurements provides complementary data that strengthen the validity of our results. Additionally, the comprehensive assessment of both periodontal and metabolic parameters allows for a more nuanced understanding of the complex interplay between local and systemic factors in periodontitis pathogenesis.

However, this study has several limitations. Its cross-sectional design prevents the identification of causal relationships between MetS and PD. Additionally, GCF sample volume was not standardized before biomarker estimation, which may have affected the quantitative results. In this study, we have excluded the periodontitis participants with CAL affecting less than 30% of the teeth, irrespective of severity. This level of CAL suggests a generalized form of periodontitis, which is suggestive of more pronounced systemic inflammatory burden. This could have affected the findings by potentially amplifying local inflammatory markers in our study. Longitudinal studies are needed to assess the predictive value of aMMP-8 expression in oral rinse and saliva for disease progression and treatment response. While we controlled for key confounding factors, such as smoking and oral hygiene habits, future research should explore the effects of medications and diet in modulating aMMP-8 levels and periodontal health.

## Conclusion

This study demonstrates that aMMP-8 serves as an effective biomarker for screening and assessing periodontitis risk, particularly in individuals with MetS. The consistent elevation of aMMP-8 above 20 ng/mL in periodontitis patients across all oral fluids supports the validity of this threshold as a diagnostic cut-off. Importantly, noninvasive oral fluids—specifically saliva and oral rinse—proved to be effective matrices for biomarker assessment, offering practical advantages over the more technically demanding GCF sampling. The significant correlations between aMMP-8 levels and both periodontal and metabolic parameters highlight the potential of this biomarker to reflect the complex interplay between local inflammation and systemic metabolic disturbances. This suggests that aMMP-8 testing could be integrated into both dental and medical screening protocols to improve early detection of PD and potentially identify individuals at risk for MetS.

In fact, recent developments in periodontitis home screening with 20 ng/mL cut-off aMMP-8 PoCT integrated with artificial intelligence-mobile applications offer promising new possibilities for patient-centered diagnostics. 27 As we move toward more personalized approaches to health care, the incorporation of noninvasive biomarker testing using readily accessible oral fluids could significantly enhance our ability to detect, monitor, and manage PD in diverse patient populations, including those with metabolic comorbidities.

## Figures and Tables

**Table:** 

1. How many times do you brush your teeth?	No brushing.	Once a day.	Twice a day or more.
2. How do you clean your teeth?	Toothbrush, fluoride toothpaste and dental floss.	Toothbrush, fluoride toothpaste.	Toothbrush only.
3. How often do you change your toothbrush?	Once in 3 months.		Once in 6 months or more.	
4. Do you use mouthwashes containing fluoride?	Often.	Sometimes.	Rare or never.
5. Do you complain of halitosis (bad smell from your mouth)?	Often.	Sometimes.	Rare or never.
6. Do you complain of bleeding on brushing or gingival bleeding?	Often.	Sometimes.	Rare or never.
7. How often do you visit the dental clinic for a check-up?	Once a year or more often.		Once every few years or when there is pain.	
8. What procedures do you do the most?	Scaling	Restorations	Extraction
9. How often do you get your teeth cleaned by a dentist?	Rare or never	Once in a year	Twice in a year

## Notes

Appendix 1. Patient Demographics, Personal Details, Oral Hygiene Status Questionnaire

1. Gender (Tick the appropriate answer)
  MaleFemale
2. Age
3. Where is your place of stay? (Tick the appropriate answer)
  Rural-urban
4. Height (cm)
5. Weight (kg)
6. Do you suffer from any systemic illness? (Tick the appropriate answer)
  No Yes specify
7. Do you take any medication on a regular for the systemic illness?
8. What is your highest educational qualification? (Tick the appropriate answer)
  No educational qualifications
  Vocational qualification
  Degree (e.g., BA, BSc)
  Postgraduate qualification
  Professional qualification
9. Do you have the habit of smoking? (Tick the appropriate answer)
  No Yes occasional
10. Are you employed? No Yes
  If yes (Tick the appropriate answer)
  - Employed Full-Time
  - Employed Part-Time
  - Seeking opportunities
  - Retired
  - Prefer not to say
